# Contribution of Food Groups to Energy and Nutrient Intakes in Five Developed Countries

**DOI:** 10.3390/nu7064593

**Published:** 2015-06-08

**Authors:** Nancy Auestad, Judith S. Hurley, Victor L. Fulgoni, Cindy M. Schweitzer

**Affiliations:** 1 Dairy Research Institute/National Dairy Council, 10255 West Higgins Road, Suite 900, Rosemont, IL 60018, USA; 2 Nutrition Insights, LLC, 44 South 2740 West, St. George, UT 84770, USA; 3 Hurley Consulting, P.O. Box 90044, Albuquerque, NM 87199, USA; E-Mail: jhurleyrd@comcast.net; 4 Nutrition Impact, LLC, 9725 D Drive North, Battle Creek, MI 49014, USA; E-Mail: vic3rd@aol.com; 5 Global Dairy Platform, 10255 West Higgins Road, Suite 820, Rosemont, IL 60018, USA; E-Mail: cindy.schweitzer@globaldairyplatform.com

**Keywords:** nutritive value, nutrition guidelines, nutritional requirements, nutritional surveys

## Abstract

Economic growth in developing countries and globalization of the food sector is leading to increasingly similar food consumption patterns worldwide. The aim of this study was to describe similarities and differences in the contributions of main food groups to energy and nutrient intakes in five developed countries across three continents*.* We obtained summary reports of national food consumption survey data from Australia, France, Denmark, the Netherlands, and the United States. Survey years spanned 2003–2012; sample size ranged from 1444 to 17,386. To mitigate heterogeneity of food groups across countries, we recategorized each survey’s reported food groups and subgroups into eight main food groups and, for three countries, a ninth “mixed dishes” group. We determined the percent contribution of each food group to mean daily intakes of energy, saturated fat, sodium, fiber, and ten vitamins and minerals that are commonly under-consumed. Differences in findings from surveys utilizing a foods-as-consumed *versus* a disaggregated or ingredients approach to food group composition and contributions from the milk and milk products group, a source of several under-consumed nutrients, were explored. Patterns of food group contributions to energy and nutrient intakes were generally similar across countries. Some differences were attributable to the analytical approach used by the surveys. For the meat/protein, milk and milk products, vegetables, and fruit groups, percent contributions to key nutrient intakes exceeded percent contributions to energy intake. The mixed dishes group provided 10%–20% of total daily energy and a similar 10%–25% of the daily intake of several nutrients. This descriptive study contributes to an understanding of food group consumption patterns in developed countries.

## 1. Introduction

Across the globe, populations vary widely in the foods they find affordable, appealing and culturally acceptable. However, economic growth in developing countries and globalization of the food sector is leading to increasingly similar food consumption patterns worldwide [1,2,3]. In addition, the prevalence of obesity and several non-communicable chronic diseases associated with obesity and diet has increased [1,2,3]. 

Many developed countries are experiencing similar diet-related population health risks and have developed national dietary guidelines to promote healthy eating patterns [4,5,6,7,8,9]. The guidelines commonly emphasize limiting energy and dietary components that are linked to increased risk for obesity, cardiovascular disease (CVD), diabetes and other non-communicable chronic diseases [4,5,6,7,8,9]. For example, guidelines of several countries recommend limiting consumption of saturated fat, *trans* fat and/or sodium to reduce risk for cardiovascular disease [4,5,6,7,8,9]. Some also advise limiting consumption of added sugars in order to lower total energy intake and improve dietary balance [4,5,6,7,8,9]. Despite these problems of over-consumption, under-consumption is also a concern. In Europe, North America and Australia, intakes of several nutrients—including vitamin C, vitamin D, folate-folic acid, calcium, iodine, potassium, selenium, and fiber—are inadequate or low [4,5,6,7,8,9,10,11]. Thus, dietary guidelines, although varying by country, generally recommend consuming more fruits, vegetables, whole grains, and low-fat or nonfat dairy products to improve the nutrient density of the diet [4,5,6,8,9]. 

Understanding patterns of food consumption and their impact on dietary balance and nutrient intake is important for planning population-based dietary guidance, particularly for developing countries experiencing economic growth and increasingly adopting Western patterns of food consumption. National dietary surveys, while having inherent limitations, provide valuable information about current food consumption patterns and the types and quantities of foods that contribute substantially to energy and nutrient intakes [12]. To holistically assess food choices in culturally different populations, we conducted a descriptive study utilizing national survey data to determine the contributions of major food groups to energy and selected nutrient intakes in five developed countries. The countries were chosen to include developed countries across three continents, as well as countries in both southern and northern Europe, given the cultural diversity across the European Union, in order to better understand similarities and differences of food group contributions to nutrients in developed countries across the globe. We also explored differences in findings from national surveys utilizing a foods-as-consumed *versus* a disaggregated or ingredients approach to food group analysis. The latter method has been selected by the European Food Safety Administration to harmonize food classification systems across the European Union [13]. This method, in which mixed dishes are disaggregated and the ingredients allocated to their respective food groups, more accurately identifies nutrient contributions from main food groups and individual foods. To date, however, not every country is using the disaggregated approach; we therefore utilized available survey reports that were based on either the foods-as-consumed or disaggregated method. 

## 2. Methods

### 2.1. Data Sources

We utilized national food consumption data from surveys conducted by government agencies in five countries: Australia, France, Denmark, the Netherlands and the United States [10,14,15,16,17]. Survey details are shown in Table 1. Survey years differed by country and spanned the period 2003–2012. Each country’s survey employed stratified, weighted sampling schemes to obtain a nationally representative sample. Sample size ranged from 1444 to 17,386. Food consumption information was obtained by 24-h dietary recall (Australia, the United States and the Netherlands) or 7-day food records (France and Denmark). 

**Table 1:** Food consumption surveys in five developed countries.

Country	Survey	Sample Population	Data Years	Dietary Data Collection	Food Group Analysis Approach
Australia	*Australian Health Survey*, Australia Bureau of Statistics [17]	Age ≥2 years, *N =* 12,153 Age 2–18 years, *N =* 2812 Age ≥19 years, *N =* 9341	2011–2012	24-h dietary recall	Foods-as-consumed
Denmark	*Danish National Survey of Diet and Physical Activity*, National Food Institute, Technical University of Denmark, Ministry of Science [10]	Age 4–75 years, *N =* 4431	2003–2008	7-day food record	Disaggregated mixed dishes
France	*Individual and National Study on Food Consumption 2 (INCA 2)*, Agence Française de Sécurité Sanitaire des Alimentes (AFFSA, French Food Safety Agency) [14]	Age 3–17 years, *N =* 1444 Age 18–79, *N =* 1918	2006–2007	7-day food record	Foods-as-consumed
The Netherlands	*Dutch National Food Consumption Survey 2007–2010*, National Institute for Public Health and the Environment, Ministry of Health, Welfare and Sport [15]	Age 7–69 years, *N =* 3819	2007–2010	24-h dietary recall	Disaggregated mixed dishes
United States	*What We Eat in America, National Health and Nutrition Examination Survey (NHANES),* Agricultural Research Service, US Department of Agriculture*;* National Center for Health Statistics, Centers for Disease Control and Prevention, US Department of Health and Human Services [16]	Age ≥2 years, *N =* 17,386 Age 2–18 years, *N =* 6090 Age ≥19 years, *N =* 11,296	2007–2008 2009–2010	24-h dietary recall	Foods-as-consumed

For our analysis, we obtained the publically available summary data reports for the surveys of Australia, France, Denmark and the Netherlands [10,14,15,16,17]. These reports provided sample-weighted mean daily intakes of energy, macronutrients and micronutrients and sample-weighted means of the percent contribution of individual foods and food groupings to the mean daily intake of energy, macronutrients, and micronutrients. For the United States, we obtained parallel estimates based on the *What We Eat in America* survey (the dietary component of the National Health and Nutrition Examination Survey), utilizing food groups as defined by the Food Surveys Research Group, United States Department of Agriculture [18,19]. Data were available for the combined population of adults and children (“total population”) in four countries (Australia, the United States, Denmark and the Netherlands) and for the separate populations of adults and children in three countries (Australia, the United States and France). Although the age ranges for the total, adult and child populations varied between countries, the majority of each country’s population was represented by the survey samples.

### 2.2. Analysis

#### 2.2.1. Food Groups

To permit cross-country comparisons, we reorganized the food categories in the survey reports from Australia (22 categories; 117 subcategories), the United States (46 categories; 150 subcategories), France (43 categories), Denmark (15 categories), and the Netherlands (17 categories; 106 subcategories) into eight main food groups: meat, poultry, fish and eggs (hereafter “meats/protein group”); milk and other dairy products (“milk and milk products group”); breads, grains and cereal products (“bread group”); vegetables; fruits; fats and oils; snacks and sweets; and “other” (Table 2). The summary data reported for Australia, France and the United States included a discrete group for mixed dishes. We retained that as a ninth food group, “mixed dishes” (described below). The reorganization resulted in overall similarity of the food items across countries in each of the eight main food groups. 

**Table 2:** Classification of foods into food groups based on national food consumption survey reports.

Food Groups ^a^	Australia	United States	France	Denmark	Netherlands
Meats, poultry, fish, eggs	Fish and seafood products; eggs; meat, poultry and game products (includes organ meats and offal; sausages; processed meat)	Meats; poultry; seafood; eggs; cured meats/poultry; plant-based protein foods (beans, peas, legumes, nuts and seeds, processed soy product)	Poultry and game; offals ^b^ meat products; fish; shellfish and mollusks; eggs and egg products;	Meat (includes offal ^b^); poultry; fish and other seafood; eggs	Meat poultry; game; organ meats and offal ^b^; processed meat; fish shellfish; eggs, egg products
Milk, other dairy products	Milk products: dairy milk (cow, sheep, goat); yoghurt; cheese; flavored milks and milkshakes; dairy and meat substitutes	Milk; cheese; yogurt; flavored milk; dairy drinks and substitutes	Milk; dairy products; cheese	Milk; flavored milk; cheese	Milk; milk beverages; yoghurt; fromage blanc; cheese; cream desserts, puddings, creamers
Breads, grains, cereal products	Flours and cereal grains; breads; English muffins; pasta; breakfast cereals	Cooked grains; breads, rolls, tortillas; quick breads and bread products; ready-to-eat cereals; cooked cereals	Breads and bread products; breakfast cereals; pasta; rice and wheat; other cereals	Bread; rice; pasta; breakfast cereals	Pasta, rice, other grain; flours; bread, crisp bread, rusks; breakfast cereals; salty biscuits, crackers; dough (puff, shortcrust, pizza)
Vegetables	Potatoes; brassica, root and stalk vegetables; peas and beans; tomatoes and tomato products; other fruiting vegetables; other vegetables	Red/orange vegetables; dark green vegetables; lettuce; corn; onions; potatoes; other starchy vegetables	Vegetables, including potatoes	Vegetables, including potatoes; ketchup	Leafy, fruiting, root and stalk vegetables; cabbages; mushrooms; onion, garlic; potatoes and other tubers
Fruits	Apples, berry, citrus, stone, tropical/subtropical and other fruit; mixtures of two or more groups of fruit; dried or preserved fruit; seed and nut products	Apples; bananas; grapes; peaches/nectarines; berries; citrus fruits; melons; dried fruits	Fruits; dried fruits, nuts and seeds; mashed and cooked fruit	Fruit (fresh and processed); dried fruit; fruit concentrates; jam; nuts and other oily seeds	Fruits, nuts and olives
Fats and oils	Butters; dairy blends; cream; margarines and table spreads; plant oils, other fats	Fats and oils (butter and animal fats; margarine; cream cheese, sour and whipped cream; cream and cream substitutes; salad dressings and vegetable oils)	Butter; oil; margarines; other fats, cream	Butter; margarine; other spreads and oils; lard; mayonnaise; tartar sauce	Vegetable oils; butter; margarines; deep frying fats; other animal fat
Snacks, sweets	Sweet and savory biscuits; cakes, muffins, scones, pastries; sugar, honey, syrups; fruit spreads; confectionary; fruit, nut and seed bars; cereal bars; potato, corn and other snacks	Sweet bakery products; candy; other desserts; sugars; honey; sugar substitutes; jams and syrups; toppings	Croissant-like pastries; biscuits (savory and sweet) bars; pastries and cakes; ice cream and iced desserts; chocolate; sugars and confectionary; cream desserts	Candy; chocolates and marzipan; honey; syrup; powdered sugar and sugar for cakes	Sugar, honey, jam; chocolate, candy bars, paste, chocolate confetti; other confectionary; syrup; ice cream; sorbet; cakes, pies, pastries; dry cakes, biscuits
Other	Non-alcoholic beverages (tea, coffee, juices, soft drinks, energy drinks, water); alcoholic beverages; sauces, dips and condiments; legumes; infant formula and foods; special dietary foods	Non-alcoholic beverages (tea, coffee, juices, soft drinks, energy drinks); alcoholic beverages; water; condiments and sauces; infant formula and baby food	Non-alcoholic beverages (includes fruit juice); alcoholic beverages; coffee; other hot drinks; waters; legumes; condiments and sauces; foods for specific needs	Coffee; tea; water; soft drinks; wine; spirits; juices; miscellaneous	Non-alcoholic beverages (fruit and vegetable juices; soft drinks; coffee; tea; waters), alcoholic beverages; legumes, condiments and sauces; soups, bouillon; dietary products; soya products
Mixed dishes ^c^	Mixed dishes where major ingredients are: cereal; fish or seafood; fruit; egg; beef, sheep, pork or mammalian game; sausage, bacon, ham or other processed meat; poultry or feathered game; milk or milk products; meat substitutes; vegetables; and soups	Meat-,poultry-, seafood- based; grain-based; Asian; Mexican; pizza; sandwiches; soups	Pizzas, salty pastries; sandwiches, hamburgers; soups; other mixed dishes	(Mixed dishes were disaggregated and each key ingredient allocated to its respective food group ^d^)	(Mixed dishes were disaggregated and each key ingredient allocated to its respective food group ^d^)

^a^ Descriptions of the food items included in the final food groups for each country follow those of the food groups and food subgroups contained in each country’s survey report; ^b^ Offal: edible, non-meat animal-based products; ^c^ Mixed dishes are multi-ingredient foods that contain combinations of two or more food groups and are consumed as a single dish; examples are pizza, sandwiches and casseroles; ^d^ For example, energy and nutrients provided by the individual food components of pizza were distributed to their respective food groups: the crust is included in the grain group, the tomato sauce in the vegetable group, the cheese in the dairy group, and the meat toppings in the meat group.

A few differences remained, as the country-specific summary datasets did not readily permit recategorization of individual food items within subgroups; however, these items made relatively small contributions to total daily energy and nutrient intakes. For example, nuts and seeds were in the fruit group for all countries except the United States, which placed them in the meats/protein group. In the United States, “nuts and seeds” accounted for 1.9% of total daily energy intakes, in Australia, “seed and nut products and dishes” accounted for 1.6% of total daily energy, and in Denmark, “dried fruit, nuts and seeds” accounted for 0.5% of total daily energy. Legumes were included in the “other” group for Australia, France and the Netherlands, where they accounted for 0.4%, 0.4% and 0.1% of daily energy, respectively. In the United States survey, legumes were included in the meats/protein group, and accounted for 0.9% of daily energy. Cream was included in the milk and milk products group for the Netherlands (where it accounted for 0.3% of daily energy), but was in the fats and oils group for Australia and the United States (where it accounted for 0.2% and 0.5% of energy respectively) and France (contribution to energy not available). 

The contributions of mixed dishes were handled differently in the summary reports of the five countries. Mixed dishes typically contained a mix of meats, dairy, grains, vegetables, or other foods (examples are pizza, hamburgers, sandwiches and casseroles). Australia, France and the United States used a “foods-as-consumed” approach in which mixed dishes constituted a separate food group in the dataset. In contrast, Denmark and the Netherlands used a “disaggregated approach” in which each mixed dish was disaggregated into its main ingredients, which were then allocated to their respective main food group. In this method, for example, the cheese in pizza is counted in the milk and milk products group, the tomato sauce in the vegetable group, and the crust in the bread group. The general process for disaggregating mixed dishes has been described by Marcoe *et al*. [20]. The approach captures more completely the contribution of a given food group to daily nutrient intakes, but requires country-specific recipe information to determine the relative proportions of food ingredients in each mixed dish. Because many mixed dishes contain meats/protein foods; milk and milk products; breads, grains and cereal products; and fats and oils, disaggregation generally results in higher energy and nutrient contributions from these food groups compared to the foods-as-consumed approach. 

#### 2.2.2. Nutrients

We summarized mean daily intakes for energy and 25 nutrients. We reviewed country-specific dietary guidelines, nutrient reference values, and other publications to identify nutrients with a higher prevalence of inadequate intake or overconsumption in the countries examined [4,6,8,9,10,11,14,21]. Based on this review, we selected 13 nutrients for inclusion in the primary analysis of food groups: fiber, vitamin A, vitamin D, folate-folic acid, vitamin B12, vitamin C, calcium, iodine, iron, potassium and selenium as nutrients commonly under-consumed, and sodium and saturated fat as nutrients commonly over-consumed.

We determined the percent contribution of the eight main food groups and mixed dishes group to mean daily intakes of energy and nutrients for the total population of four countries. We also calculated food group contributions to daily nutrient intakes for the separate adult and child populations for Australia (adults age ≥19 years; children age 2–18 years), France (adults age ≥18 years; children age 3–17 years) and the United States (adults age ≥19 years; children age 2–18 years). In addition, we used selected nutrients as examples to graphically illustrate similarities and differences in food group nutrient contributions in different countries and to compare food group contributions to nutrient intakes in countries using the foods-as-consumed approach and the disaggregated approach. The nutrients selected include two over-consumed nutrients (saturated fat and sodium) and three under-consumed nutrients (calcium, vitamin B12 and vitamin C). For these example nutrients, we identified food groups providing more than 10% of the daily intake in one or more countries; when a food group provided more than 10% of the nutrient’s intake for any one country, that food group was included for all countries in the data graph.

Because the milk and milk products group is an important source of several nutrients of interest and its nutrient contributions are partially obscured in surveys that use the foods-as-consumed approach, we examined its role in the diets of adults and children in three countries more closely, focusing on nutrients that are commonly under- or overconsumed and for which milk and milk products are important contributors: saturated fat, vitamin A, vitamin B12, vitamin D, calcium, iodine, potassium, selenium and sodium [22]. 

## 3. Results

For reference purposes, the mean daily energy and nutrient intakes in Australia, the United States and the European countries of France, Denmark and the Netherlands are provided in Table 3. In countries with total population data available (adults and children combined), mean daily energy intake ranged from a low of 8493 kilojoules (kJ) (2030 kcals) in Australia to a high of 9510 kJ (2273 kcals) in the Netherlands, a difference of 12%. Across these same countries, mean nutrient intakes differed by 30% or more (from lowest to highest) for fiber, calcium, iron, potassium, selenium, sodium, and vitamins A, B1, B2, B6, D, E and folate.

**Table 3:** Mean intake of energy and nutrients in five developed countries.

	Australia (*N =* 12,153)	United States (*N =* 17,386)	France ^1^		Denmark (*N =* 4431)	Netherlands (*N =* 3819)
			Adults (*N =* 1918)	Children (*N* =1444)		
Age of sample population, years	≥2	≥2	18–79	3–17	4–75	7–69
Energy, kilojoules (kilocalories)	8493 (2030)	8686 (2076)	9046 (2162)	7431 (1776)	8891 (2125)	9510 (2273)
Protein, g	88	79	87	68	74	80
Total fat, g	73	78	89	75	80	83
Saturated fatty acids, g	28	26	36	32	34	31
Monounsaturated fatty acids, g	28	28	32	26	28	29
Polyunsaturated fatty acids, g	11	17	13	10	12	15
Carbohydrate, g	229	258	229	207	242	254
Fiber, g	22	16	18	13	20	19
Vitamin A:						
Retinol, µg	313	-	702	445	741	642
RAE, µg	815	617	-	-	1093	827
Vitamin D, µg	-	5	2.6	1.9	3.1	3.4
Vitamin E, µg	10	7.4	11.5	9.2	7	13.4
Vitamin B1, mg	1.6	1.6	1.2	1.1	1.2	1.2
Vitamin B2, mg	1.9	2.1	1.9	1.6	1.6	1.6
Vitamin B6, mg	1.4	2.0	1.7	1.5	1.4	2.0
Folate-folic acid:						
Folate, µg	278	397	287	227	304	-
DFE, µg	613	534	-	-	-	268
Vitamin B12, µg	4.4	5.2	5.8	3.9	5.1	4.7
Vitamin C, mg	100	85	93	77	105	97
Calcium, mg	805	987	914	807	1053	1052
Iodine, µg	172	-	126	106	189	176
Iron, mg	10.8	14.8	13.1	10.2	9.7	10.2
Magnesium, mg	320	283	292	211	334	349
Phosphorus, mg	1422	1342	1267	1063	1377	1544
Potassium, mg	2800	2576	2969	2264	3200	3382
Selenium, µg	86	-	53	38	41	47
Sodium, mg	2404	3462	2968	2146	3300	2711
Zinc, mg	10.6	11.6	10.7	8.3	10.3	10.6

Abbreviations: DFE, dietary folate equivalent; RAE, retinol activity equivalent; Dash (-), data not available; ^1^ Total population data for France not available.

### 3.1. Total Population

The contributions of the eight main food groups and the mixed dishes group (where relevant) to intakes of energy and selected nutrients in the total population of four countries are shown in Table 4. Countries using the foods-as-consumed approach are shown in the upper portion of the table and countries using the disaggregated approach in the lower portion. In Australia and the United States, countries utilizing foods-as-consumed food groups, the meats/protein group accounted for 11%–16% of daily energy, 14%–22% of saturated fat and 15%–22% of sodium intake, along with more than one-quarter of daily vitamin B12 and, in Australia, 30% of daily selenium. The milk and milk products group contributed 8% of daily energy and about one-fifth of daily saturated fat, along with 12%–22% of the vitamin A, 22%–30% of the vitamin B12, 36%–40% of the calcium and, in the United States, 49% of the vitamin D. Breads, grains and cereal products contributed 14%–18% of daily energy, 23%–29% of daily fiber, 42%–46% of folate-folic acid, about one-third of daily iron and, in Australia, nearly one-third of daily iodine. Vegetables contributed 5% of total daily energy and 13%–29% of daily fiber, vitamin A and vitamin C. The fruit group (as defined in Table 2), contributed 3%–6% of daily energy, 12%–17% of daily fiber and 17%–23% of vitamin C (fruit juice, contained in the “other” group, provided an additional 16%–31% of vitamin C in these two countries; data not shown). Fats and oils, excluding those used in mixed dishes, contributed 2%–3% of energy intake and made negligible contributions to intakes of under-consumed nutrients. Snacks and sweets contributed 17% of daily energy and 18%–26% of saturated fat in both countries. Mixed dishes contributed about one-fifth of daily energy and saturated fat and ≥20% of daily fiber, vitamin B12, iron, and sodium. 

Because mixed dishes tend to include meats, dairy, grains and fats/oils and these ingredients have been allocated to their respective food groups in the surveys from Denmark and the Netherlands, the results for these countries indicate generally higher energy and nutrient contributions from those food groups than were seen in countries using the foods-as-consumed approach, although the meat group’s contribution to energy intake is highest in the United States, a country that used the foods-as-consumed approach. 

### 3.2. Adult and Child Populations

The percentages of daily energy and nutrients contributed by food groups in adults and children are shown in Table 5a,b, respectively. The three countries with data available for these population groups utilized the foods-as-consumed approach. The percentages of daily energy that adults derive from individual food groups were remarkably similar across Australia, the United States and France (Table 5a). The relative energy contributions of food groups were also quite similar for children across countries (Table 5b). Some differences existed, however. In both adults and children, energy intake from meats/protein foods was somewhat lower in Australia than in the United States and France, while energy intake from mixed dishes was somewhat lower in France than in the other two countries (10% *versus* 18%–21%). In French children, energy and nutrient intakes from the milk and milk products group were similar to those of children in Australia and the United States, with the exception of vitamin D, for which the milk and milk products group accounted for only 17% of total daily intake (France does not fortify milk with vitamin D, as the United States does; vitamin D data for Australia were not available). In France, the bread group contributions to daily energy and fiber intakes in children and adults were similar to Australia and the United States, but contributions to folate-folic acid, iodine, iron and selenium were lower. In the three countries, vegetables contributed 4%–6% of daily energy and 10%–30% of daily fiber, vitamin C and potassium; however, in France, vegetables contributed a notably lower proportion of vitamin A intake and higher proportion of folate-folic acid intake.

In comparing energy and nutrient intakes of adults and children within a single country, some differences are apparent. In Australia, the United States, and France, sweets and snacks contributed a greater proportion of total energy and saturated fat intake in the diets of children than in adults. Adults obtained larger percentages of energy, vitamin B12, iron and selenium intakes from the meats/protein group than did children. However, the milk and milk products group contributed greater proportions of the daily intakes of energy and several nutrients (vitamin A, vitamin D, vitamin B12, calcium, iodine and potassium) in children’s diets.

**Table 4:** Contribution of food groups to total daily energy and nutrient intakes in four developed countries, total population.

Food Group	Percent contribution to total daily intake (%)													
	Energy	Fiber	Saturated Fat	Vitamin A	Vitamin D	Vitamin B9 ^a^	Vitamin B12	Vitamin C	Calcium	Iodine	Iron	Potassium	Selenium	Sodium
**Countries using foods-as-consumed analysis methods (8 main food groups and 1 mixed dishes group)**														
**Australia, age ≥2 years**														
Meats, poultry, fish, eggs	11	2	14	7	-	3	31	0	3	7	14	11	30	15
Milk, other dairy products	8	1	19	12	-	6	30	1	40	26	2	13	5	8
Breads, grains, cereal products	18	29	4	1	-	46	4	1	13	28	31	9	15	18
Vegetables	5	16	4	29	-	6	0	20	3	2	8	15	2	2
Fruits	6	17	2	5	-	5	0	23	3	0	5	11	5	0
Fats and oils	2	0	7	6	-	0	0	0	0	0	0	0	0	1
Snacks, sweets	17	9	26	8	-	4	4	2	9	6	10	8	6	14
Other	14	7	5	7	-	15	8	36	15	16	9	15	11	13
Mixed dishes	19	20	21	25	-	13	22	16	15	15	22	19	26	29
**United States, age ≥2 years**														
Meats, poultry, fish, eggs	16	9	22	8	22	6	27	1	5	-	15	17	-	22
Milk, other dairy products	8	1	18	22	49	2	22	1	36	-	2	12	-	7
Breads, grains, cereal products	14	23	4	14	8	42	15	3	12	-	36	6	-	15
Vegetables	5	16	4	17	1	7	2	18	4	-	5	13	-	7
Fruits	3	12	0	2	0	2	0	17	1	-	2	7	-	0
Fats and oils	3	0	7	5	1	0	1	0	1	-	0	1	-	3
Snacks, sweets	17	13	18	9	2	10	4	4	8	-	12	9	-	9
Other	15	5	2	7	6	7	6	49	16	-	7	18	-	9
Mixed dishes	20	21	24	17	10	23	22	9	18	-	22	17	-	30
**Countries using disaggregated analysis methods (8 main food groups, no separate mixed dishes group)**														
**Denmark, age 4–75 years ^b^**														
Meats, poultry, fish, eggs	14	0	21	38	78	10	56	7	3	9	26	15	56	-
Milk, other dairy products	15	1	30	13	12	16	37	3	60	37	3	18	19	-
Breads, grains, cereal products	28	53	6	2	4	21	0	0	8	18	32	12	16	-
Vegetables	7	23	3	29	0	30	0	42	5	2	18	24	3	-
Fruits	7	20	1	2	0	14	0	30	3	1	6	11	2	-
Fats and oils	11	0	32	15	5	0	6	0	0	0	1	0	0	-
Snacks, sweets	7	1	6	1	0	0	1	0	4	1	5	2	1	-
Other	12	2	2	3	10	10	1	20	18	34	13	23	12	-
**Netherlands, age 7–69 years ^b^**														
Meats, poultry, fish, eggs	13	2	20	17	34	7	44	10	4	9	20	15	43	21
Milk, other dairy products	14	4	30	23	5	11	38	4	58	15	3	17	14	16
Breads, grains, cereal products	23	43	6	1	0	22	0	1	8	53	26	12	17	31
Vegetables	6	23	2	15	0	21	0	29	6	3	13	20	4	3
Fruits	5	11	2	1	0	5	0	16	2	1	4	7	4	1
Fats and oils	7	0	14	21	36	7	3	0	1	0	0	0	0	1
Snacks, sweets	15	9	17	7	8	3	3	2	6	5	13	6	5	6
Other	16	9	7	8	8	14	7	28	14	12	17	22	8	21

^a^ Vitamin B9 values includes both folate (the form of vitamin B9 naturally present in foods) and folic acid (the synthetic form of vitamin B9 used in food fortification); ^b^ Mixed dishes are disaggregated and the main ingredients allotted to their respective food groups.

**Table 5:** (**a**) Contribution of foods groups and mixed dishes to total daily energy and nutrient intakes in three developed countries, adult population; (**b**) Contribution of foods groups and mixed dishes to total daily energy and nutrient intakes in three developed countries, child population.

(a)														
Food Group	Percent contribution to total daily intake (%)													
	Energy	Fiber	Saturated Fat	Vitamin A	Vitamin D	Folate-Folic Acid	Vitamin B12	Vitamin C	Calcium	Iodine	Iron	Potassium	Selenium	Sodium
**Australia, age ≥19 years**														
Meats, poultry, fish, eggs	11	2	16	8	-	3	34	0	3	8	15	12	32	16
Milk, other dairy products	8	1	18	10	-	6	27	1	38	24	2	11	4	7
Breads, grains, cereal products	18	29	4	1	-	46	3	1	12	28	30	9	14	18
Vegetables	5	17	3	30	-	7	0	22	4	1	8	15	2	2
Fruits	6	17	2	5	-	5	0	22	3	0	6	10	6	0
Fats and oils	2	0	7	6	-	0	0	0	0	0	0	0	0	1
Snacks, sweets	16	8	24	7	-	3	4	2	8	6	9	7	5	12
Other	15	7	5	7	-	15	9	35	17	18	10	16	12	14
Mixed dishes	19	20	21	26	-	14	22	17	15	15	22	19	25	29
**United States, age ≥19 years**														
Meats, poultry, fish, eggs	17	10	23	9	27	7	30	1	6	-	16	17	-	23
Milk, other dairy products	7	1	16	19	43	2	19	1	33	-	1	10	-	6
Breads, grains, cereal products	13	23	4	13	8	41	14	3	12	-	34	6	-	14
Vegetables	6	17	5	19	2	8	2	20	4	-	6	14	-	7
Fruits	3	11	0	2	0	2	0	16	1	-	2	7	-	0
Fats and oils	3	0	8	5	1	0	2	0	1	-	0	1	-	3
Snacks, sweets	16	12	17	9	2	9	4	3	8	-	22	8	-	8
Other	16	5	3	7	7	8	7	46	18	-	8	19	-	9
Mixed dishes	20	21	24	17	11	23	23	9	18	-	22	17	-	29
**France, age 18–79 years**														
Meats, poultry, fish, eggs	15	0	18	55	57	11	67	3	5	26	25	18	46	19
Milk, other dairy products	10	1	22	15	13	12	14	2	43	26	3	11	8	10
Breads, grains, cereal products	19	27	2	0	1	15	1	2	7	9	15	7	12	27
Vegetables	5	25	2	0	2	24	0	30	6	5	11	19	3	4
Fruits	4	18	0	0	2	10	0	29	2	5	4	10	1	0
Fats and oils	9	0	22	13	4	0	0	0	0	0	0	0	1	1
Snacks, sweets	17	10	21	9	8	8	3	3	9	6	13	7	7	7
Other	11	7	3	1	3	11	2	25	19	11	17	18	11	11
Mixed dishes	10	12	11	6	12	9	13	7	10	13	12	12	11	21
Dash (-), data not available														
**(b)**														
**Food Group**	**Percent contribution to total daily intake (%)**													
	**Energy**	**Fiber**	**Saturated Fat**	**Vitamin A**	**Vitamin D**	**Folate-Folic Acid**	**Vitamin B12**	**Vitamin C**	**Calcium**	**Iodine**	**Iron**	**Potassium**	**Selenium**	**Sodium**
**Australia, age 2–18 years**														
Meats, poultry, fish, eggs	8	2	11	2	-	2	22	0	2	4	10	9	24	13
Milk, other dairy products	11	1	24	19	-	7	42	1	47	34	2	18	7	9
Breads, grains, cereal products	18	29	5	1	-	48	5	2	13	30	35	8	19	20
Vegetables	5	13	3	28	-	4	0	14	2	1	6	13	1	2
Fruits	6	18	1	5	-	5	0	26	2	1	5	11	2	0
Fats and oils	2	0	5	6	-	0	0	0	0	0	0	0	0	1
Snacks, sweets	23	13	32	11	-	5	5	4	11	8	13	12	10	17
Other	9	6	2	6	-	15	3	42	9	10	9	11	8	9
Mixed dishes	18	18	18	20	-	13	21	11	13	13	20	17	28	27
****United States, age 2–18 years****														
Meats, poultry, fish, eggs	13	7	18	5	11	5	19	1	4	-	11	14	-	20
Milk, other dairy products	12	3	23	32	67	3	33	1	45	-	3	22	-	9
Breads, grains, cereal products	14	22	5	19	9	47	20	4	12	-	40	6	-	16
Vegetables	4	12	3	10	1	4	1	9	2	-	4	10	-	5
Fruits	3	13	0	2	0	2	0	17	1	-	2	7	-	0
Fats and oils	2	0	5	3	1	0	1	0	0	-	0	0	-	2
Snacks, sweets	20	15	19	9	1	11	4	5	7	-	14	10	-	11
Other	12	4	2	4	4	4	4	57	12	-	5	13	-	7
Mixed dishes	21	23	25	15	7	25	19	6	18	-	22	17	-	30
**France, age 3–17 years**														
Meats, poultry, fish, eggs	13	1	15	40	47	10	52	2	3	21	20	16	40	18
Milk, other dairy products	12	1	20	22	17	12	22	4	49	34	5	19	11	11
Breads, grains, cereal products	16	25	2	0	1	19	4	6	8	6	19	6	11	21
Vegetables	5	23	2	1	1	18	0	22	5	5	10	17	2	5
Fruits	3	13	0	0	0	7	0	20	1	1	3	8	1	0
Fats and oils	7	0	17	14	4	0	0	0	0	0	0	0	1	1
Snacks, sweets	25	18	30	14	15	13	6	2	13	16	22	12	13	13
Other	8	9	2	1	2	13	2	39	11	7	10	12	9	10
Mixed dishes	10	12	10	8	14	9	15	5	9	11	11	11	11	23
Dash (-), data not available														

### 3.3. Foods-as-Consumed *versus* Disaggregated Method

**Figure 1:**
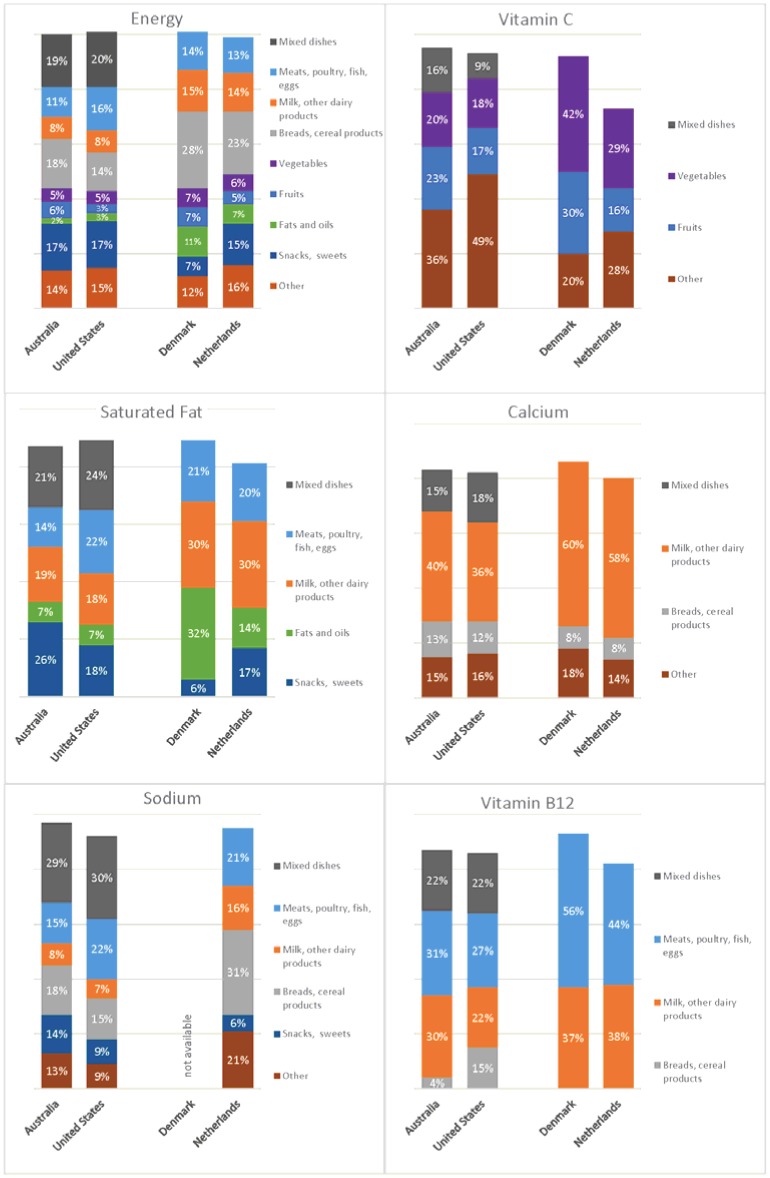
Percent contribution of food groups to daily energy and selected nutrient intakes in the total population in countries using the foods-as-consumed (Australia and the United States) and disaggregated (Denmark and the Netherlands) analyses methods (total population data for France not available). Countries using the disaggregated approach have eight food groups; countries using the foods-as-consumed approach have eight food groups plus a ninth mixed dishes group. For energy, percent nutrient contributions from all food groups are shown (bars may not total 100% due to rounding); for other nutrients, a food group is shown if it provided more than 10% of the daily intake in at least one country.

Figure 1 graphically compares the contribution of food groups to energy and selected nutrient intakes for countries using the foods-as-consumed approach (Australia and the United States) and those using the disaggregated approach (Denmark and the Netherlands). Food groups contributing more than 10% of the daily intake of the nutrient in at least one country are shown. In the disaggregated approach, the ingredients in mixed dishes have been spread across the main eight food groups, with correspondingly greater percent nutrient contributions from those groups. For example, it can be seen that the contributions from the bread group to energy intake and of the vegetable group to vitamin C intake were notably higher in Denmark and the Netherlands. These differences are not necessarily entirely due to the food group analysis method; they may in part represent real differences in food consumption patterns between countries. A striking example of the differences produced by the different analysis methods is the milk and milk products group. It contributed 8% of daily energy in Australia and the United States, but nearly twice that in Denmark (14%) and the Netherlands (15%). The milk and milk products group’s contribution to calcium intake rose from 40% and 36% in Australia and the United States, respectively, to 60% and 58% in Denmark and the Netherlands. These differences highlight the impact of food grouping methods on the interpretation of the relative nutritional importance of individual foods in the diet and the difficulty of comparing food consumption patterns across countries that use different methods of food group categorization.

**Figure 2:**
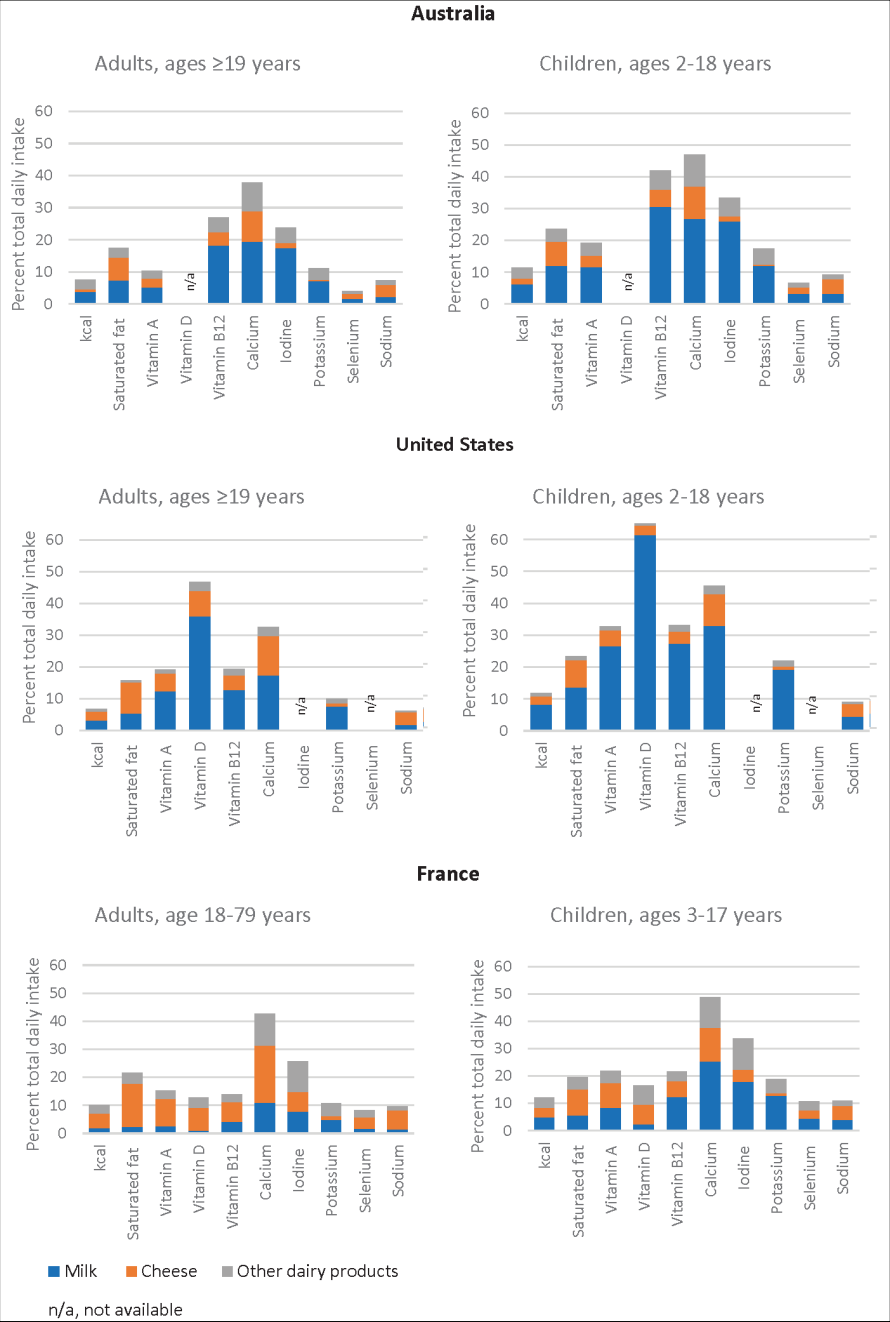
Contribution of milk, cheese and other dairy products consumed as individual foods to energy and selected nutrient intakes in children and adults in Australia, the United States and France.

Because the milk and milk products group is an important source of several nutrients of interest and its nutrient contributions are partially obscured in surveys that use the foods-as-consumed approach, we examined its role in the diets of adults and children in three countries more closely (Figure 2). The nutrients in Figure 2 were selected from the nutrients we identified as commonly under- or over-consumed and for which milk and other dairy products are important contributors.

Dairy foods, although contributing to saturated fat intake, made important contributions to daily intakes of calcium, vitamin A and vitamin D (in the United States) and to iodine (in Australia and France) in both age groups, even when not considering dairy ingredients in mixed dishes. Milk in particular made noteworthy contributions to daily intakes of calcium (11%–33%), potassium (5%–19%) and iodine (8%–26%). In the United States, due to fortification, milk also made important contributions to intakes of vitamin A (12%–27%) and vitamin D (36%–61%). In France, cheese accounted for a greater proportion of the daily intake of several nutrients than it did in Australia or the United States. In all three countries, milk’s contributions to daily intakes of several under-consumed nutrients were higher in children than in adults. 

## 4. Discussion

Using national food consumption data, we identified the contributions of a broad range of food groups to intakes of energy and 13 nutrients in five developed countries across three continents: Australia, North America and Europe. In these countries, the main food groups each contributed remarkably similar proportions of daily energy and nutrient intakes for most of the over- and under-consumed nutrients examined. Similarities are seen in the data for the total population as well as for the separate populations of children and adults. For the meat/protein, milk and milk products, vegetables, and fruit groups, percent contributions to key nutrient intakes exceeded percent contributions to energy intake, reflecting the nutrient density of these food groups.

We found expected differences in nutrient contributions from food groups based on the analytical approach used by the surveys, *i.e.*, the foods-as-consumed approach *versus* the disaggregated or ingredients approach, in which the components of mixed dishes such as pizza, sandwiches and casseroles are separated and allocated to their respective main food groups. These differences serve to highlight the difficulty of comparing food consumption trends across countries that use different methods of food group analysis and the need for harmonization of food survey methods, as recommended by the European Food Safety Administration [13].

Dietary patterns of developed countries, and to a lesser extent of developing countries, are converging [1,2,3]. One aspect of that convergence is over-consumption of certain dietary components that are associated with increased risk for non-communicable chronic diseases. At the same time, the identification of specific food choices within food groups and of specific dietary patterns, such as the Mediterranean diet pattern, that reduce chronic disease risk is an increasingly active area of research [23]. While this evaluation describes only broad patterns of food group consumption and their contribution to nutrient intakes, it reveals surprisingly consistent patterns across countries with different historic and cultural food habits. To our knowledge, this is the first study to examine similarities and differences in food group contributions to nutrient intakes in several developed countries on different continents. A similar approach would also be valuable for understanding dietary patterns in developing countries, where food habits are rapidly changing [1,2,3].

Across the globe, population-based dietary guidance typically calls for moderation of energy intake to achieve and maintain a healthy weight; lower intakes of saturated fat and sodium; increased consumption of fruits, vegetables, whole grains and low-fat or nonfat dairy products; and higher intakes of under-consumed nutrients. While underreporting of energy intake is a well-recognized phenomena in dietary surveys, we found total daily mean energy intake to be remarkably similar in the five countries studied (8593–9501 kJ or 2030–2274 kcal), only a 12% difference across countries. Saturated fat intake varied across countries from 11.2% to14.9% of energy. These values are remarkably consistent with those identified in a review of dietary fat intake in 40 countries (11.0%–15.0% of energy for the five countries of interest) [24]. Four of the eight main food groups (plus the mixed dishes group, where used) accounted for 80 percent or more of daily saturated fat intake. The Denmark and Netherlands analyses, which used the disaggregated approach, showed greater contributions of the fats/oils and milk and milk products groups to saturated fat intakes and of the milk and milk products group to the intakes of several under-consumed nutrients (calcium, iodine, potassium, selenium, and vitamin B12). This is consistent with previous studies from Europe and the United States [22,25]. Of note, recent reviews indicate that not all food sources of saturated fat are associated with increased risk for CVD [26,27,28,29,30,31,32,33]. Several studies indicate that dairy consumption is not associated with CVD risk and some suggest dairy may contribute to a reduction in CVD [27,28,29,30,31,32,33,34,35]. An independent association between intake of milk and milk products and body weight has not been demonstrated [6]. Consumption of milk and milk products continues to be encouraged in many countries [32]. Global and national dietary guidance also call for reductions in sodium intake. The World Health Organization recommends that sodium intake not exceed 2000 mg/day for adults in order to reduce risk for hypertension, CVD, stroke and coronary heart disease [36]. However, questions have been raised in recent reviews of sodium intake and the ratio of sodium to potassium in diets across the globe [37,38,39]. In four of the five countries we studied, sodium intake in adults ranged from 2711 to 3462 mg/day (daily intake was lower in Australia, where sodium intake from salt added at the table was not included in survey data). These intakes are within the 2645–4945 mg/day range that was associated with the most favorable health outcomes and no increase in mortality in a meta-analysis of 23 cohort and two follow-up studies (*N =* 274,683) [38].

Country-specific fortification of some foods is reflected in our study findings. In the United States, where virtually all milk is fortified with vitamin D, daily vitamin D intake was higher than in the three European countries that do not fortify milk (5 µg/day *versus* 2.6–3.4 µg) (the greater contribution of the meat/protein group to vitamin D intake in Denmark and France was consistent with higher fish consumption in those countries) [40]. Similarly, higher folate-folic acid intake in the United States and Australia is likely explained by the mandatory fortification of some flour with folic acid in these countries. 

This study has several limitations. First, the sampling methods, sample sizes, and survey years varied by country. However, the samples were nationally representative of each country’s population and the data reports provided sample-weighted means of nutrient intakes by specific food groups, allowing for reasonable comparisons across countries. All data collection occurred across the ten-year period 2003–2012. It is unlikely that temporal changes in food consumption during this time were substantial enough to affect between-country comparisons. According to a synthesis of food consumption data from 127 countries, global intake of saturated fat was stable between 1990 and 2010, increasing only 0.3% in Australia and decreasing between 0.1% and 0.9% in the other countries that we studied [41]. Second, the food consumption data of each country were obtained using 24-h dietary recall or 7-day dietary records kept by participants. Although a single 24-h dietary recall does not capture the variation that is present in an individual’s diet, both 24-h dietary recalls and 7-day diet records are validated methods for surveying dietary intake in large populations and estimating group mean intakes of foods and nutrients. The limitations of dietary records and recall methods, including the underreporting of energy intake (primarily due to underestimation of portion sizes by participants), have been widely documented and discussed in the literature [12,42,43]. In 24-h dietary recall facilitated by trained interviewers, for example, participants may not accurately report dietary intake for a variety of reasons related to “knowledge, memory or the interview situation” [12]. However, there is no reason to assume that any inherent limitations of these dietary data collection methods applied more to one country than another. Third, methods for processing food consumption data into food groups varied by country, as each incorporated a food group/food coding scheme relevant to its national diet. However, we recategorized food groupings into a standard number of food groups, which minimized differences across countries in the composition of the final food groups. Another limitation is that different food composition databases underlie the nutrient calculations provided in each country’s summary data reports, so that nutrient values for the same food or type of food may differ slightly from country to country [3]. These minor differences are unlikely to have had a significant impact on the analysis.

## 5. Conclusions

Food consumption survey data from five developed countries showed that major food groups contribute remarkably similar percentages of the daily intakes of energy, saturated fat, fiber and selected nutrients. Our analysis also highlights differences in food group nutrient contributions that may be attributable to the method of food group analysis used rather than to differences in food consumption patterns *per se*. This analysis provides new information useful to understanding food group consumption patterns in developed countries, an important task given the increasing convergence of dietary habits across the globe, and highlights methodological considerations relevant to future cross-country studies of dietary sources of nutrients.
